# A fundus image dataset for intelligent diabetic retinopathy system

**DOI:** 10.1038/s41597-026-07093-7

**Published:** 2026-04-01

**Authors:** Shaojuan Peng, Shuo Yang, Xinyu Zhao, Yongtao Zhang, Qingjie Bai, Duo Yuan, Yaling Liu, Yarou Hu, Yi Chen, Kaixuan Cui, Zhen Yu, Zhenquan Wu, Ruyin Tian, Baiying Lei, Guoming Zhang

**Affiliations:** 1 Shenzhen Eye Hospital, Shenzhen Eye Center, Southern Medical University, Guangzhou, China; 2 National-Regional Key Technology Engineering Laboratory for Medical Ultrasound, Guangdong Key Laboratory for Biomedical Measurements and Ultrasound Imaging, College of Biomedical Engineering, Shenzhen University Medical School, Shenzhen University, Shenzhen, China; 3 The First Affiliated Hospital of Shandong First Medical University & Shandong Provincial Qianfoshan Hospital, Jinan, China

**Keywords:** Retinal diseases, Vision disorders

## Abstract

Diabetic retinopathy (DR), the most prevalent microvascular complication of diabetes mellitus, is the leading cause of irreversible vision loss in the global working-age population. At present, deep learning-integrated ultra-wide-field (UWF) image analysis systems have improved DR grading consistency and reduced peripheral lesion misdiagnosis rates, thereby overcoming the limitations of traditional 45° viewing field AI models in detecting peripheral retinal lesions. However, the lack of standardized, high-quality, and publicly available UWF-DR datasets has severely restricted the generalization ability and reliability of AI models in clinical practice. To address this, this study constructed a dataset comprising 1,630 UWF fundus images from 809 patients, which were annotated and classified by three senior ophthalmologists, for development and validation of AI system in UWF-based DR diagnosis. This dataset aims to empower researchers to train more efficient and accurate AI-assisted DR diagnosis systems based on UWF images, advancing its widespread real-world clinical applications.

## Background & Summary

Diabetic retinopathy (DR), a hallmark microvascular complication of diabetes^1–3^, is characterized by retinal vascular damage and remains the leading cause of irreversible vision loss among the global working-age population^4,5^. According to the International clinical DR (ICDR) guidelines^1^, the severity of DR is categorized as:1. No apparent retinopathy 2. Non-proliferative DR (NPDR), further subclassified into mild, moderate, and severe stages 3. Proliferative DR (PDR)^1^. Diabetic macular edema (DME), can occur at any stage of NPDR or PDR^6,7^. Its core mechanism is capillary leakage in the macular area, which causes retinal thickening and central vision damage^8^. Early-stage DR is typically asymptomatic, necessitating glycemic control and regular screenings (e.g., fundus photography; UWF imaging device)^9^. In PDR, fibrovascular membrane contraction may cause tractional retinal detachment (TRD)^10^, while anterior chamber angle invasion by neovessels leads to neovascular glaucoma (NVG)^11^—both are vision-threatening complications. The World Health Organization (WHO) emphasizes systematic fundus screening as a cost-effective strategy to reduce DR-related blindness^12^, particularly in resource-limited clinical settings^13^. Therefore, early diagnosis and intervention of DR through regular fundus examination and systematic fundus screening is an important strategy to reduce the blindness rate caused by DR^1,7^.

**Fig. 1:**
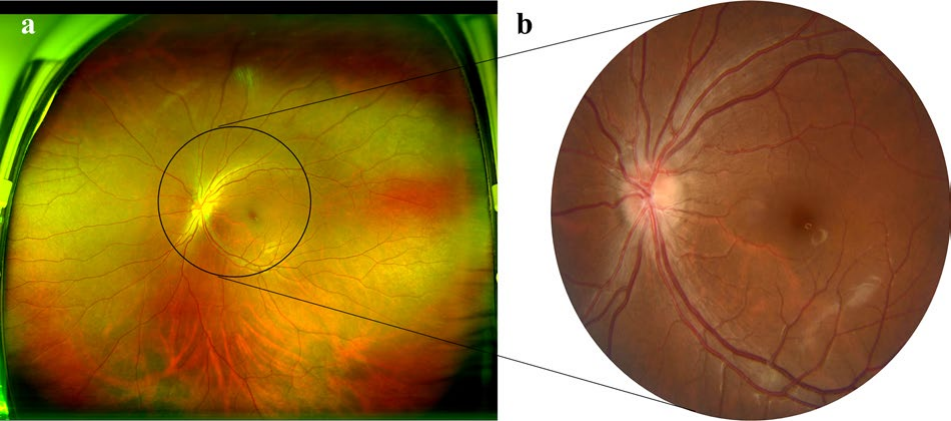
The difference in visual field coverage between ultra-wide-field fundus photography and traditional narrow-field fundus photography (45° viewing field). (**a**) ultra-wide-field fundus photography. (**b**) 45° viewing field fundus photography.

In recent years, the integration of retinal imaging technology and artificial intelligence (AI) has significantly advanced the process of automated diagnosis of DR^14^.Current research focuses on automatic DR detection and grading systems based on traditional narrow-field fundus photography (45° viewing field)^15,16^, but its limited field of view leads to a misdiagnosis rate of peripheral retinal lesions as high as 46%^17^. Notably, the emerging UWF fundus imaging technology covers up to 200° viewing field retinal area, approximately five times the range of traditional viewing field^18,19^, as shown in Fig. 1. Its wide-field characteristics can not only significantly improve the detection rate of peripheral ischemic lesions and neovascularization^20^, but also assist precise laser treatment decisions through quantitative assessment of peripheral areas^21–23^. The UWF system achieves multi-dimensional evaluation from anatomical structure to functional metabolism^24^, providing a new technical paradigm for accurate grading and personalized diagnosis and treatment of DR^25^. Although UWF imaging technology holds clinical potential, a review of the literature available through 2022 demonstrates that only a very small number of studies based on AI have been conducted (23/4358)^26^. In addition, although publicly available UWF fundus image datasets were relatively scarce in early studies^27^, recent multi-center collaborations and technological innovations have significantly advanced the construction and sharing of high-quality UWF datasets^28^. For instance, Peking Union Medical College Hospital (PUMCH) developed a UWF-CKDS dataset of UWF images to support the training of a chronic kidney disease screening model^29^. In another study, the team proposed the ASModel_UWF based on 11,528 UWF images for anemia screening^30^. The Ningbo Institute of Materials team propose an unsupervised lesion-aware transfer learning framework, which based on the joint training of 904 annotated UWF images and traditional fundus images, realizing the automatic grading of DR^31^. Based on this, the high-quality DR UWF dataset we established will also help improve the generalization ability and clinical applicability of AI models in DR grading, and promote the transformation of AI-based DR screening technology from laboratory research to multicenter clinical trials and real-world applications^28^.

**Fig. 2:**
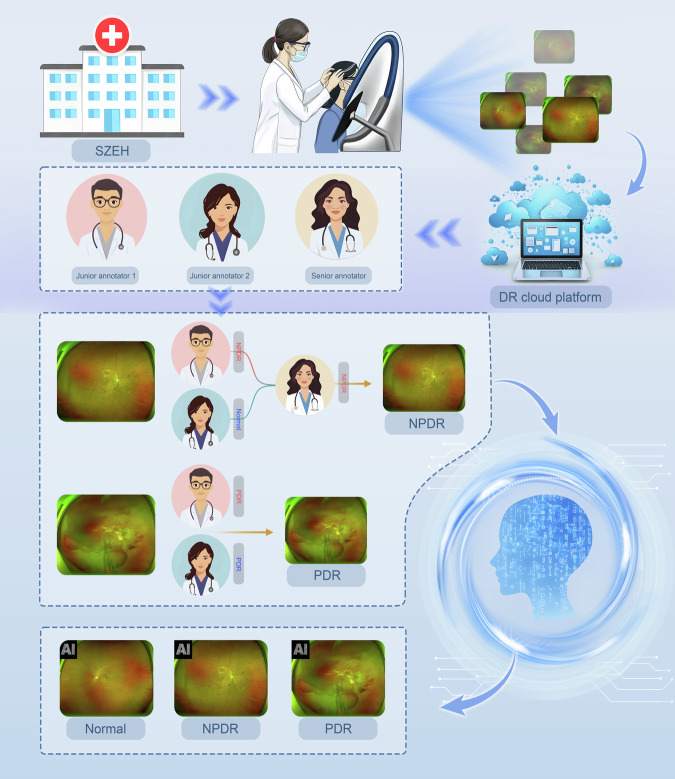
DR dataset establishment process: 1. Data collection hospitals. 2. A total of 1630 UWF images were collected and uploaded into the DR cloud platform. 3. Three annotators who had completed guideline-based training and image annotation tests were included in image classification task. Two junior annotators perform preliminary annotations and one senior annotator performs further verification. 4. The final classification results were used to develop and validate the AI model for automated DR screening.

In this study, we provided 1630 fundus images of normal, NPDR and PDR, adopted four models to develop and validate them at the same time. It is necessary to be noted that the classification outcome of each image may still exhibit discrepancies compared to judgments by different ophthalmologists globally, even after implementing standardized protocols. Despite all the ophthalmologists adhere to the same ICDR criteria for fundus image classification, divergent conclusions may arise due to the subjective nature of visual assessment. Crucially, the staging inconsistency between normal and NPDR manifests most significantly among annotators. A brief description of this study is shown in Fig. 2. We hope that this high-quality fundus image dataset can be used by researchers to train more efficient, accurate, and clinically applicable AI-assisted DR diagnosing systems based on UWF images. More importantly, the combination of UWF images and AI diagnosing systems features “wide coverage^32^, high precision, low manpower, and easy deployment”^33^. It is conducive to optimizing the cost-effectiveness of systematic DR screening in resource-limited clinical environments and will bring revolutionary changes to the medical field^34^.

## Methods

### Data collection

A total of 1630 UWF fundus images were collected from 2018 to 2023 at Shenzhen Eye Hospital (SZEH) and Shenmei Eye Hospital. All participants in this dataset are Asian (Han Chinese), with a mean age of 53.8 ± 12.1 years, and males account for 63.87% (1041/1630). Informed consent was waived due to the retrospective nature of the study. All images were de-identified prior to the analysis to protect privacy. This study adhered to the tenets of the Helsinki Declaration and was approved by the Medical Ethics Committee of SZEH (ID: 2025KYPJ080). Images in this study were captured by Optomap Daytona. The image acquisition process was as follows: patients did not need to dilate their pupils before the examination. The patient took a sitting position with the head fixed on the forehead and chin rests of the device to ensure that the eyeballs were aligned with the scanning light path and kept their eyes open naturally. Fundus photography was performed by trained technicians or ophthalmologists. The operator adjusted the device and used the automatic shooting function to automatically capture when the fixation light turned green. By guiding the patient to rotate to multiple eye positions (up, down, left, right, center), combined with the automatic stitching technology of the device, the imaging range was expanded to 220°–240° viewing field for complete imaging.

We first excluded images with poor quality, such as severe artifacts, out-of-focus, blur, and incomplete images, to ensure clear visibility of the lesions. The images finally included need to clearly show the retina, blood vessels, and lesion features and be considered clinically acceptable and usable.

Due to the progressive nature of DR, the same patient may be followed up multiple times, so the same eye may contain multiple fundus images. All selected images were exported in JPG format. We applied preprocessing techniques to uniformly crop all images to a resolution of 256 × 256 pixels, in order to standardize the input data and improve computational efficiency during model training. All images have been fully uploaded to Figshare^35^. These files are available for researchers to perform advanced feature extraction, detailed lesion analysis, and other subsequent processing.

### Image classification

**Fig. 3:**
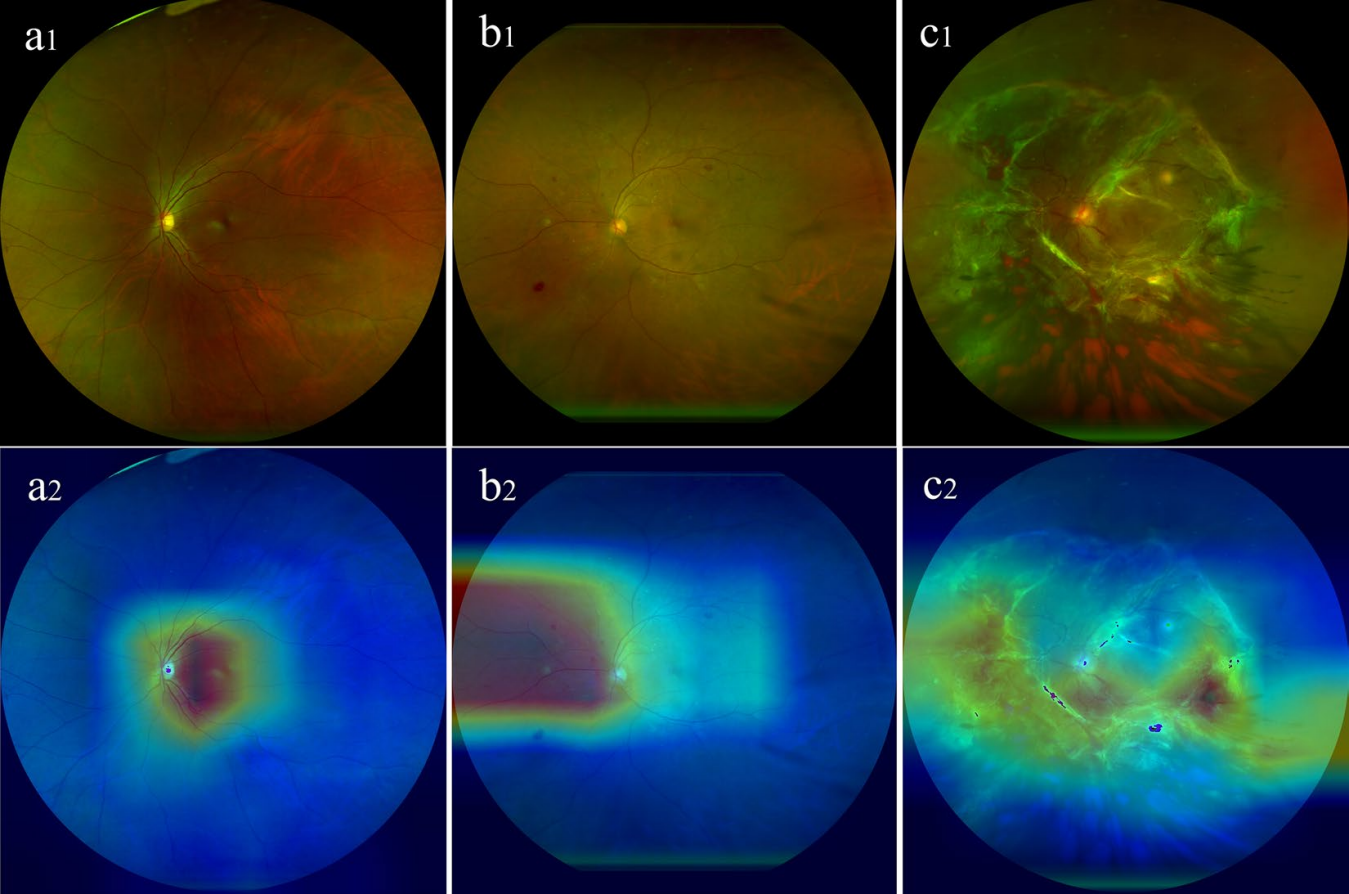
Heatmaps for demonstration of typical features for each category. (**a1**) Normal image; (**a2**) Heatmap of normal image (**b1**), Non-proliferative diabetic retinopathy (NPDR) image; (**b2**) Heatmap of NPDR image (**c1**), Proliferative diabetic retinopathy (PDR) image; (**c2**) Heatmap of PDR image.

According to the ICDR guidelines, all UWF fundus images were classified into three categories (Table 1): normal, NPDR, PDR. Representative images of each category of fundus images are shown in Fig. 3. The images were classified by three expert annotators from SZEH to ensure accuracy. Heatmap visualizations highlighted the characteristic regions of interest, revealing key pathological features such as microaneurysms and proliferative retinal membranes. These findings support the model’s accuracy in identifying clinically relevant areas.

**Table 1:** The distribution of UWF fundus images for three categories.

Data	Train set	Validation set	Test set	Total images	Patients
Normal	347	50	99	496	204
NPDR	444	63	127	634	344
PDR	350	50	100	500	261
Total	1141	163	326	1630	809

## Data Records

The dataset, titled “Ultra-wide-field (SLO) fundus image dataset for intelligent diabetic retinopathy system”, is provided as a zipped file^35^. The main folder “diabetic retinopathy” contains three subfolders named “Normal”, “NPDR” and “PDR”, alongside three XLS worksheets: test.xls, train.xls and validate.xls. The “Normal” subfolder contains 512 × 512 pixel fundus images without any signs of DR. The “NPDR” subfolder contains 512 × 512 pixel fundus images of non-proliferative DR. The “PDR” subfolder contains 512 × 512 fundus images of proliferative DR. The three XLS worksheets list image filenames and their corresponding labels for the test, training, and validation sets, respectively. Core variables in these worksheets include image name (the filename of the fundus image) and label (the name of the subfolder to which the fundus image belongs, where 0 represents normal, 1 represents NPDR, and 2 represents PDR). This dataset has not been previously published or made available elsewhere in any other form and is publicly available on https://figshare.com/. It can be used for automated identification, localization, and lesion grading of DR, as well as the validation of DR-related AI models developed by multiple researchers.

## Technical Validation

**Fig. 4:**
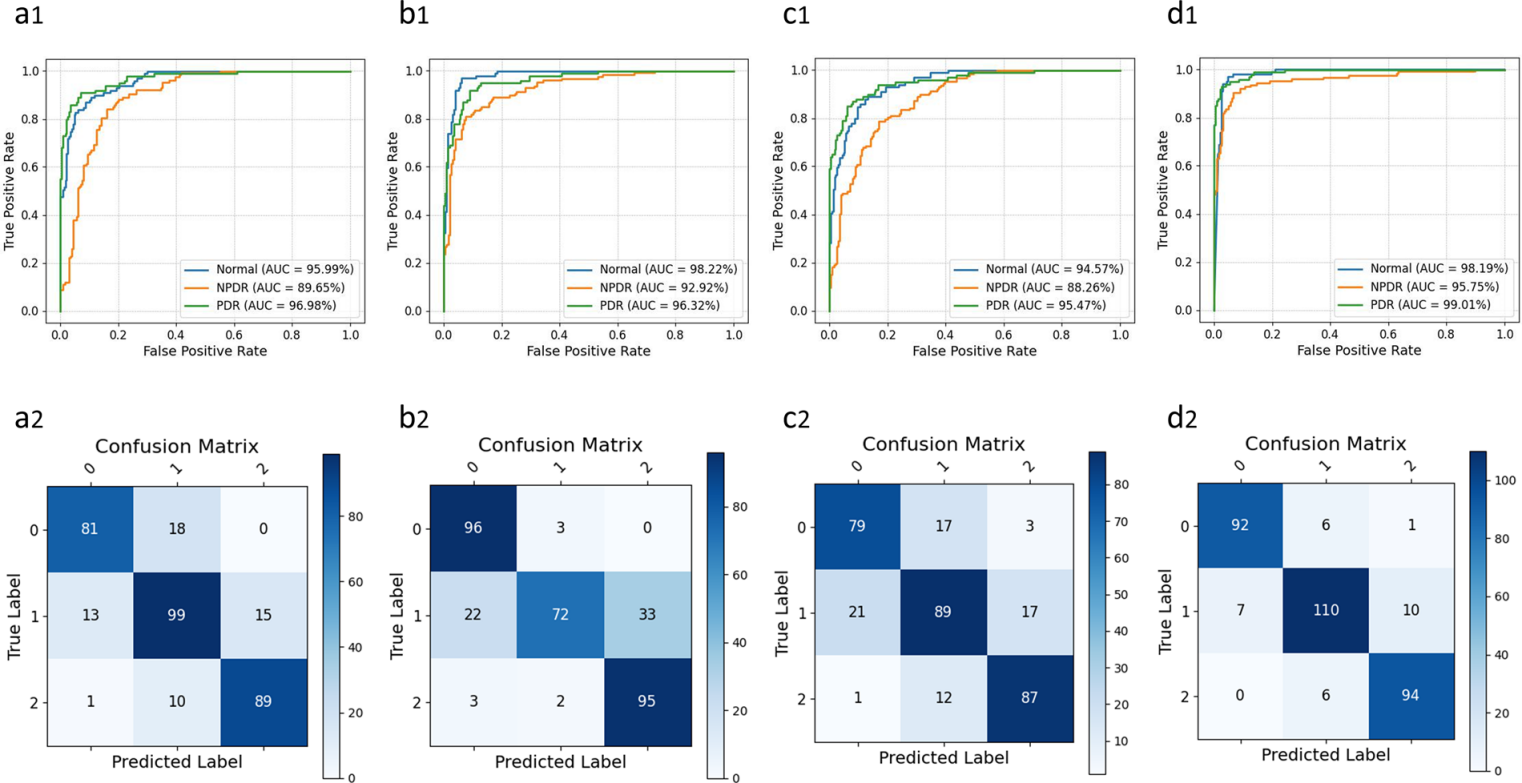
The classification results based on different AI models. (**a**–**d**) display the performance of the ResNet34 model, DenseNet121 model, InceptionV4 model and Xception model.

To evaluate the utility of the dataset in training and testing AI models, we developed four deep learning models to automatically classify DR based on UWF fundus images. In order to make the public dataset more suitable for the training and validation of artificial intelligence models, this study selectively screened fundus images with clear lesion characteristics, thereby publishing a high-quality labelled dataset. The dataset was divided into training, validation, and test sets in a ratio of 7:1:2. During training, we adopted some common data augmentation strategies, including random crop, random horizontal and vertical flipping, etc. The stochastic gradient descent optimizer was used to optimize the parameters of the AI models. The learning rate was set to 0.00125 and the cosine algorithm was used to update it. All of the AI models’ training cycles were set to 500 epochs, and the batch size was set to 16. All of the AI models were implemented on an NVIDIA GeForce RTX 4090. Four algorithms (ResNet34, DenseNet121, InceptionV4 and Xception) were selected for the development and validation. The performance of each model was evaluated using a range of metrics, including accuracy (ACC), area under the receiver operating characteristic curve (AUC), precision (PRE), sensitivity (SEN), specificity (SPE), F1 score, and Kappa value (Table 2). The classification results demonstrated excellent performance across these indicators. Moreover, as shown in Fig. 4, the correspondence between the prediction of the AI model and the actual classification is clearly displayed through the confusion matrix, which facilitates the comprehensive evaluation of the performance of various models and error analysis. The classification results on the test dataset show that all AI models can achieve excellent performance in the classification task. Among them, the AI model developed by the Xception network achieved the best performance. These results support the technical quality of the DR dataset.

**Table 2:** The classification results of four AI models.

Method	ACC (%)	AUC (%)	PRE (%)	SEN (%)	SPE (%)	F1 (%)	Kappa (%)
ResNet34	82.52	94.21	82.93	82.92	91.06	82.90	84.92
DenseNet121	80.67	95.82	82.35	82.89	91.31	80.40	83.93
InceptionV4	78.22	92.77	78.32	78.96	88.97	78.57	79.61
Xception	90.80	97.65	90.87	91.18	95.29	91.00	91.81

## Usage Notes

We encourage users to cite the figshare record and this article and acknowledge the contribution of this dataset in their studies.

## Data Availability

The dataset is publicly available at Figshare^35^ (10.6084/m9.figshare.31259494).

## Code availability

No novel code was used in the construction of the dataset.

## References

[1] Flaxel, C. J. *et al*. Diabetic Retinopathy Preferred Practice Pattern®. *Ophthalmology* **127**(1), P66–p145 (2020). 31757498 10.1016/j.ophtha.2019.09.025

[2] Xu, R. *et al*. Molecular mechanism and intervention measures of microvascular complications in diabetes. *Open Med (Wars)* **19**(1), 20230894 (2024). 38645437 10.1515/med-2023-0894 PMC11032097

[3] Muc, R., Saracen, A. & Grabska-Liberek, I. Associations of Diabetic Retinopathy with Retinal Neurodegeneration on the Background of Diabetes Mellitus. Overview of Recent Medical Studies with an Assessment of the Impact on Healthcare systems. *Open Med (Wars)* **13**, 130–136 (2018). 29675479 10.1515/med-2018-0008 PMC5906647

[4] Kropp, M. *et al*. Diabetic retinopathy as the leading cause of blindness and early predictor of cascading complications-risks and mitigation. *Epma j* **14**(1), 21–42 (2023). 36866156 10.1007/s13167-023-00314-8 PMC9971534

[5] Yau, J. W. *et al*. Global prevalence and major risk factors of diabetic retinopathy. *Diabetes Care* **35**(3), 556–64 (2012). 22301125 10.2337/dc11-1909 PMC3322721

[6] Wilkinson, C. P. *et al*. Proposed international clinical diabetic retinopathy and diabetic macular edema disease severity scales. *Ophthalmology* **110**(9), 1677–82 (2003). 13129861 10.1016/S0161-6420(03)00475-5

[7] Wong, T. Y. *et al*. Guidelines on Diabetic Eye Care: The International Council of Ophthalmology Recommendations for Screening, Follow-up, Referral, and Treatment Based on Resource Settings. *Ophthalmology* **125**(10), 1608–1622 (2018). 29776671 10.1016/j.ophtha.2018.04.007

[8] Omar, A. *et al*. Diabetic Disease of the Eye in Canada: Consensus Statements from a Retina Specialist Working Group. *Ophthalmol Ther* **13**(5), 1071–1102 (2024). 38526804 10.1007/s40123-024-00923-0 PMC11039592

[9] Oluleye, T. current management of diabetic maculopathy. *Journal of Diabetes and Metabolism*, **3** (2011).

[10] Mishra, C. & Tripathy, K. *Retinal Traction Detachment*, in *StatPearls*. StatPearls Publishing Copyright © 2025, StatPearls Publishing LLC.: *Treasure Island (FL) ineligible companies*. Disclosure: Koushik Tripathy declares no relevant financial relationships with ineligible companies (2025).

[11] Mishra, C. & Meyer, J. J. *Neovascular Glaucoma*, in *StatPearls*. StatPearls Publishing Copyright © 2025, StatPearls Publishing LLC.: Treasure Island (FL) ineligible companies. Disclosure: Jay Meyer declares no relevant financial relationships with ineligible companies (2025).

[12] Huemer, J., Wagner, S. K. & Sim, D. A. The Evolution of Diabetic Retinopathy Screening Programmes: A Chronology of Retinal Photography from 35 mm Slides to Artificial Intelligence. *Clin Ophthalmol* **14**, 2021–2035 (2020). 32764868 10.2147/OPTH.S261629 PMC7381763

[13] Chabba, N. *et al*. What is the coverage of retina screening services for people with diabetes? Protocol for a systematic review and meta-analysis. *BMJ Open* **14**(1), e081123 (2024). 38296278 10.1136/bmjopen-2023-081123 PMC10828834

[14] Hassan, B. *et al*. A comprehensive review of artificial intelligence models for screening major retinal diseases. *Artificial Intelligence Review* **57**(5), 111 (2024).

[15] Huang, X. *et al*. Artificial intelligence promotes the diagnosis and screening of diabetic retinopathy. *Front Endocrinol (Lausanne)* **13**, 946915 (2022). 36246896 10.3389/fendo.2022.946915 PMC9559815

[16] Van Craenendonck, T. *et al*. Systematic Comparison of Heatmapping Techniques in Deep Learning in the Context of Diabetic Retinopathy Lesion Detection. *Transl Vis Sci Technol* **9**(2), 64 (2020). 33403156 10.1167/tvst.9.2.64 PMC7774113

[17] Pearce, E. & Sivaprasad, S. A Review of Advancements and Evidence Gaps in Diabetic Retinopathy Screening Models. *Clin Ophthalmol* **14**, 3285–3296 (2020). 33116380 10.2147/OPTH.S267521 PMC7569040

[18] Balyen, L. Ultra-widefield imaging technologies in the peripheral retinal pathologies. *Exploration of Medicine* **4**(1), 1–2 (2023).

[19] Midena, E. *et al*. Ultra-wide-field fundus photography compared to ophthalmoscopy in diagnosing and classifying major retinal diseases. *Sci Rep* **12**(1), 19287 (2022). 36369463 10.1038/s41598-022-23170-4 PMC9650656

[20] Peng, Y. *et al*. Enhancing AI reliability: A foundation model with uncertainty estimation for optical coherence tomography-based retinal disease diagnosis. *Cell Rep Med* **6**(1), 101876 (2025). 39706192 10.1016/j.xcrm.2024.101876 PMC11866418

[21] Chen, Y. *et al*. Regional assessment of choroidal vascularity index in patients with pre- and early-stage diabetic retinopathy using ultra-wide-field OCTA. **11** (2024). 10.3389/fmed.2024.1490831 PMC11540705 39512617

[22] Soomro, T. *et al*. Recent advances in imaging technologies for assessment of retinal diseases. *Expert Rev Med Devices* **17**(10), 1095–1108 (2020). 32885710 10.1080/17434440.2020.1816167

[23] Shin, Y. *et al*. Comparison between Deep-Learning-Based Ultra-Wide-Field Fundus Imaging and True-Colour Confocal Scanning for Diagnosing Glaucoma. 2022. **11**(11): p. 3168. 10.3390/jcm11113168 PMC9181263 35683577

[24] Li, J. *et al*. Ultra-widefield color fundus photography combined with high-speed ultra-widefield swept-source optical coherence tomography angiography for non-invasive detection of lesions in diabetic retinopathy. *Front Public Health* **10**, 1047608 (2022). 36408020 10.3389/fpubh.2022.1047608 PMC9667033

[25] Gawęcki, M. & Kiciński, K. Advantages of the Utilization of Wide-Field OCT and Wide-Field OCT Angiography in Clinical Practice. *Diagnostics (Basel)*, **14**(3) (2024). 10.3390/diagnostics14030321 PMC10855083 38337837

[26] Tang, Q. Q. *et al*. Applications of deep learning for detecting ophthalmic diseases with ultrawide-field fundus images. *Int J Ophthalmol* **17**(1), 188–200 (2024). 38239939 10.18240/ijo.2024.01.24 PMC10754665

[27] Wu, R. *et al*. Automatic Segmentation of Hemorrhages in the Ultra-wide Field Retina: Multi-scale Attention Subtraction Networks and An Ultra-wide Field Retinal Hemorrhage Dataset. *IEEE J Biomed Health Inform*, **Pp** (2024). 10.1109/JBHI.2024.3457512 39255077

[28] He, S. *et al*. Open ultrawidefield fundus image dataset with disease diagnosis and clinical image quality assessment. *Scientific Data* **11**(1), 1251 (2024). 39567563 10.1038/s41597-024-04113-2 PMC11579006

[29] Zhao, X. *et al*. Screening chronic kidney disease through deep learning utilizing ultra-wide-field fundus images. *NPJ Digit Med* **7**(1), 275 (2024). 39375513 10.1038/s41746-024-01271-w PMC11458603

[30] Zhao, X. *et al*. Deep-Learning-Based Hemoglobin Concentration Prediction and Anemia Screening Using Ultra-Wide Field Fundus Images. *Front Cell Dev Biol* **10**, 888268 (2022). 35663399 10.3389/fcell.2022.888268 PMC9160874

[31] Chen, T. *et al*. Cross-modality transfer learning with knowledge infusion for diabetic retinopathy grading. *Front Med (Lausanne)* **11**, 1400137 (2024). 38808141 10.3389/fmed.2024.1400137 PMC11130363

[32] Engelmann, J. *et al*. Detecting multiple retinal diseases in ultra-widefield fundus imaging and data-driven identification of informative regions with deep learning. *Nature Machine Intelligence* **4**(12), 1143–1154 (2022).

[33] Wei, X. *et al*. MSTNet: Multi-scale spatial-aware transformer with multi-instance learning for diabetic retinopathy classification. *Med Image Anal* **102**, 103511 (2025). 40020421 10.1016/j.media.2025.103511

[34] Purohit, N. *et al*. Optimizing Diabetic Retinopathy Screening at Primary Health Centres in India: A Cost-Effectiveness Analysis. *Pharmacoecon Open*, (2025). 10.1007/s41669-025-00572-4 PMC12209073 40205319

[35] Peng, S.-J. *et al*. Ultra-wide-field (SLO) fundus image dataset for intelligent diabetic retinopathy system. *figshare*10.6084/m9.figshare.31259494 (2026). 10.1038/s41597-026-07093-7 PMC13201777 41922360

